# An eHealth Intervention for Promoting COVID-19 Knowledge and Protective Behaviors and Reducing Pandemic Distress Among Sexual and Gender Minorities: Protocol for a Randomized Controlled Trial (#SafeHandsSafeHearts)

**DOI:** 10.2196/34381

**Published:** 2021-12-10

**Authors:** Peter A Newman, Venkatesan Chakrapani, Charmaine Williams, Notisha Massaquoi, Suchon Tepjan, Surachet Roungprakhon, Pakorn Akkakanjanasupar, Carmen Logie, Shruta Rawat

**Affiliations:** 1 Factor-Inwentash Faculty of Social Work University of Toronto Toronto, ON Canada; 2 Centre for Sexuality and Health Research and Policy Chennai India; 3 Department of Health and Society University of Toronto Scarborough Scarborough, ON Canada; 4 VOICES-Thailand Foundation Chiang Mai Thailand; 5 Faculty of Science and Technology Rajamangala University of Technology Phra Nakhon Bangkok Thailand; 6 The Humsafar Trust Mumbai India

**Keywords:** COVID-19, eHealth, RCT, protective behaviors, psychological distress, LGBT+, India, Thailand

## Abstract

**Background:**

Existing data on COVID-19 disparities among vulnerable populations portend excess risk for lesbian, gay, bisexual, transgender (LGBT) and other persons outside of heteronormative and cisgender identities (ie, LGBT+). Owing to adverse social determinants of health, including pervasive HIV and sexual stigma, harassment, violence, barriers in access to health care, and existing health and mental health disparities, sexual and gender minorities in India and Thailand are at disproportionate risk for SARS-CoV-2 infection and severe disease. Despite global health disparities among LGBT+ populations, there is a lack of coordinated, community-engaged interventions to address the expected excess burden of COVID-19 and public health–recommended protective measures.

**Objective:**

We will implement a randomized controlled trial (RCT) to evaluate the effectiveness of a brief, peer-delivered eHealth intervention to increase COVID-19 knowledge and public health–recommended protective behaviors, and reduce psychological distress among LGBT+ people residing in Bangkok, Thailand, and Mumbai, India. Subsequent to the RCT, we will conduct exit interviews with purposively sampled subgroups, including those with no intervention effect.

**Methods:**

SafeHandsSafeHearts is a 2-site, parallel waitlist-controlled RCT to test the efficacy of a 3-session, peer counselor–delivered eHealth intervention based on motivational interviewing and psychoeducation. The study methods, online infrastructure, and content were pilot-tested with LGBT+ individuals in Toronto, Canada, before adaptation and rollout in the other contexts. The primary outcomes are COVID-19 knowledge (index based on US Centers for Disease Control and Prevention [CDC] items), protective behaviors (index based on World Health Organization and US CDC guidelines), depression (Patient Health Questionnaire-2), and anxiety (Generalized Anxiety Disorder-2). Secondary outcomes include loneliness, COVID-19 stress, and intended care-seeking. We will enroll 310 participants in each city aged 18 years and older. One-third of the participants will be cisgender gay, bisexual, and other men who have sex with men; one-third will be cisgender lesbian, bisexual, and other women who have sex with women; and one-third will be transfeminine, transmasculine, and gender nonbinary people. Participants will be equally stratified in the immediate intervention and waitlist control groups. Participants are mainly recruited from online social media accounts of community-based partner organizations. They can access the intervention on a computer, tablet, or mobile phone. SafeHandsSafeHearts involves 3 sessions delivered weekly over 3 successive weeks. Exit interviews will be conducted online with 3 subgroups (n=12 per group, n=36 in each city) of purposively selected participants to be informed by RCT outcomes and focal populations of concern.

**Results:**

The RCT was funded in 2020. The trials started recruitment as of August 1, 2021, and all RCT data collection will likely be completed by January 31, 2022.

**Conclusions:**

The SafeHandsSafeHearts RCT will provide evidence about the effectiveness of a brief, peer-delivered eHealth intervention developed for LGBT+ populations amid the COVID-19 pandemic. If the intervention proves effective, it will provide a basis for future scale-up in India and Thailand, and other low- and middle-income countries.

**Trial Registration:**

ClinicalTrials.gov NCT04870723; https://clinicaltrials.gov/ct2/show/NCT04870723

**International Registered Report Identifier (IRRID):**

DERR1-10.2196/34381

## Introduction

### Background

As of September 30, 2021, India reported 33,739,980 COVID-19 cases and 448,062 deaths [1], and Thailand [2] reported 1,603,475 cases and 16,727 deaths [3,4]. In India, the vast informal workforce, poverty, food insecurity, and an underfunded health care system make projections difficult [5]. Thailand faces similar socioeconomic challenges but on a lesser scale. World Bank [6,7] data reveal that India (0.9/1000) and Thailand (0.8/1000) have nearly 3-fold lower ratios of physicians per capita compared with those of the United States and Canada (both 2.6/1000), with a 5-fold lower ratio of hospital beds in India (0.5/1000) compared with those of the United States (2.9/1000), Canada (2.5/1000), and Thailand (2.1/1000). These data indicate serious health care system challenges amid unknown third and fourth waves of infections and emerging variants of concern [8].

Sexual and gender minorities in India and Thailand, as in many contexts, face pervasive HIV and sexual stigma, harassment, violence, and barriers in access to health care [9-15]. In India, national HIV prevalence among men who have sex with men (MSM) and transgender people is 20-fold higher than that of the general population [16-18]. Depression and alcohol dependence incidence is 5-fold higher among lesbian, gay, bisexual, and transgender (LGBT) people than that among the general population [19-21]. Thailand has a 10-fold higher HIV prevalence among MSM versus the general population [22]. Limited research indicates that depression among LGBT adults is ~10-fold higher than that of the general population [23,24]. High rates of suicidal ideation and substance use have been documented among transgender people [25,26], lesbian women [27,28], MSM [29,30], and LGBT youth [31].

The ongoing pandemic along with various forms of lockdowns and stay-at-home orders in India and Thailand amid new waves of COVID-19 pose particular threats to LGBT and other persons outside of heteronormative and cisgender identities (LGBT+) populations [5,32]. Existing data on COVID-19 disparities among vulnerable populations [33,34] portend an excess risk for LGBT+ people. Mental health challenges due to public health–recommended protective measures (eg, masking, physical distancing) [35], stay-at-home orders, and community-based organization (CBO) closures threaten excess risks for LGBT+ people compounded by stigma and related stress [36,37]. Social and structural vulnerabilities among LGBT+ people that are associated with existing mental health disparities are likely to be exacerbated due to the trauma and social isolation of the pandemic [38], including increases in depression, anxiety, and loneliness [35,39-42].

Adverse social determinants of health [43] (SDOH), including unstable housing, marginal employment, and discrimination in health care, impact the ability to enact physical distancing, work from home, and access testing [33,35]. LGBT+ people, who are more vulnerable owing to adverse SDOH [38,44-48], are among the populations at excess risk, along with people living with HIV [49], ethnic and racial minorities [31], and immigrants and refugees [31,50]. The gendered impacts of the pandemic, including women's disproportionate responsibilities for informal care within families and employment on the frontlines of health care [51], also intersect with these other vulnerabilities [52]. Populations in low- and middle-income countries (LMICs), including LGBT+ people in particular, face risks exacerbated by structural challenges and lack of human rights protections [5,53].

Despite pervasive global health disparities among LGBT+ populations, adverse SDOH [54-56], and lack of human rights protections [32,57], there is a lack of coordinated, community-engaged responses to address the expected excess burden of COVID-19 and public health–recommended protective measures. Public health responses and communications for LGBT+ communities are impeded by lack of LGBT+ community engagement, along with lack of data on health disparities and community needs in the pandemic. Lessons learned from Ebola, H1N1 influenza, and SARS [58,59] indicate that engaging vulnerable communities and building trust are crucial to public health communication and responses [58-61]. With pandemic planning typically framed around the traditional nuclear family and stereotypical gender role assumptions [58], public health–recommended measures often overlook LGBT+ people with different living configurations than those of heterosexual, cisgender people: living with same-gender partners/spouses, friends, hostile families, or alone. This compounds vulnerabilities due to social isolation, and lack of social support and safety [58,62]. Undifferentiated public health responses can exacerbate mistrust among vulnerable communities due to existing disparities, fueling loss of confidence in public health communications [58,59] in a broader context of rampant COVID-19 misinformation [63].

Some restrictions on rights are justified in response to a public health emergency. Nevertheless, as reported by Human Rights Watch [64], UNAIDS (Joint United Nations Programme on HIV/AIDS) [65], the Office of the United Nations High Commissioner for Human Rights [66], and the media [67,68], government emergency powers in response to COVID-19 have led to abuses against LGBT+ people worldwide. These include housing discrimination, evictions, and police brutality against transgender people in India [67]; disproportionate job loss and lack of access to government subsidies for LGBT+ people in Thailand and India, many of whom are marginalized from the mainstream workforce [42]; and exacerbation of sexual and HIV stigma [65,66].

In sum, heightened vulnerability among LGBT+ populations in the COVID-19 pandemic may result from existing health disparities amid ongoing adverse SDOH, compounded by human rights violations and social-structural constraints on enacting public health–recommended protective measures. Yet, public health responses largely do not address LGBT+ vulnerabilities. Extensive evidence supports the acceptability [36,69] and effectiveness of eHealth interventions with LGBT+ and other vulnerable populations in increasing health knowledge and preventive behaviors, and reducing psychological distress [70,71]. Our World Health Organization (WHO)-recommended approach based on community engagement [72] in intervention development, capacitation of CBOs, and cogovernance by trusted CBO partners supports the feasibility, acceptability, and scalability of the intervention [60,73]. The proposed #SafeHandsSafeHearts intervention aims to support LGBT+ individuals amid the pandemic and to advance the broader pandemic response for LGBT+ populations.

### Research Questions

This study addresses the following research questions: What are the needs and challenges faced by diverse LGBT+ people in India and Thailand in the COVID-19 pandemic? What is the level of COVID-19 knowledge, public health–recommended protective behaviors, and psychological distress? Will a brief, tailored, peer-led eHealth intervention increase COVID-19 knowledge and protective behaviors, and reduce psychological distress among LGBT+ people?

### Specific Objectives

The specific objectives of the study are to (1) increase knowledge about COVID-19 transmission, risk, and public health–recommended protective behaviors among diverse LGBT+ persons in India and Thailand; (2) increase public health–recommended protective behaviors, including handwashing, physical distancing, and wearing masks; and (3) reduce pandemic-related psychological distress (anxiety, depression, social isolation/loneliness).

## Methods

### Ethics Approval and Consent to Participate

Ethics approvals have been received from the University of Toronto Research Ethics Board (RIS Protocol: 39769); the Humsafar Trust Institutional Review Board (Protocol: HST-IRB-51-06/2021); and the Institutional Review Board of the Faculty of Medicine, Chulalongkorn University (Protocol: 272/64).

### Study Design

We use a sequential quantitative-qualitative mixed methods design [74]. We will implement a 2-site, parallel waitlist-controlled randomized controlled trial (RCT) to test the efficacy of a 3-session, peer counselor–delivered eHealth intervention to increase COVID-19 knowledge and protective behaviors, and decrease pandemic-related psychological distress. The immediate versus waitlist allocation ratio is 1:1. We conducted a formative intervention development phase with LGBT+ individuals in Toronto to pilot test and refine the online study infrastructure (participant eligibility screening, randomization, survey programming and administration, databases, and dashboard interface for tracking and monitoring) and peer counselor training before rollout in the other 2 sites. All study materials were culturally and contextually adapted through consultation with experts in each site, pilot-tested among individuals from locally eligible study populations, and then revised before implementation.

The immediate intervention group to be enrolled over a 2-month period will complete the 3-session biweekly intervention from August to October 2021. The randomized waitlist control group will crossover to receive the intervention from October to December 2021, after the immediate group finishes. Waitlist control groups, often used in psychosocial interventions [75], avert ethical problems with no-treatment controls, particularly with groups that experience health disparities, moreover amid a pandemic, and also avoid alienating the community.

### Sample Size Calculations

The sample size was calculated based on power to detect significant differences in 3 primary outcomes: proportion of participants with: (1) accurate COVID-19 knowledge, (2) consistent public health–recommended handwashing behaviors, and (3) pandemic-related psychological distress. Given baseline differences in the two countries, we first describe the detailed power analysis for India, where (1) COVID-19 knowledge ranges from 18.2% (fever a major symptom) to 43.0% (highly contagious) [76], and (2) a national survey [77] estimated that 35.8% of the population wash their hands with soap and water. Our baseline estimates are 40% for knowledge and 36% for handwashing (proxy for 3 protective behaviors). A 30% increase postintervention attains clinical/public health significance [78] and a substantial effect size. For (3) psychological distress (depression, anxiety, social isolation), we use a baseline depression rate of 50% (based on a systematic review [19]) as a proxy, with an expected 30% reduction [79]. Using Stata-16, the required sample size to detect significant differences between the waitlist control and immediate-intervention groups, with power of 80%, α of .05 for the 95% CI, and a two-tailed test, ranged from 78 to 86. Assuming 20% attrition, the final sample size was increased to 103 per group (cisgender men, cisgender women, transgender and gender nonbinary people) with power to detect significant differences in each of the 3 groups, for a total sample of 309 in Mumbai.

Using published prevalence of primary outcomes and estimated effect sizes based on similar in-country interventions, we estimated a sample size of 309 for Bangkok [23,28,80-82]. Thus, the trial is powered to detect overall (and by city) sex and gender differences in the primary outcomes (COVID-19 knowledge, protective behaviors, and psychological distress) and the efficacy of the intervention [83].

### Procedures

#### Inclusion Criteria

Participants are eligible for enrollment if they are (1) aged 18 years and older; (2) self-identify as LGBT+ using local, culturally appropriate self-identifications [10,57,79]; (3) reside in one of the two cities (Bangkok and Mumbai); (4) able to understand and willing to provide informed consent; and (5) able to understand primary language(s) at the site (Thai, Hindi/Marathi, or English). We do not use exclusion criteria based on mental health. The Patient Health Questionnaire-2 (PHQ-2) will be administered in the baseline survey; those with scores indicative of clinical depression (≥3 on the depression scale) will be referred by peer counselors to in-house mental health professionals on call at the site.

#### Recruitment

Participants will be recruited online with electronic flyers and social media messages developed with CBO partners, through CBO social media accounts in WhatsApp groups, e-groups, virtual LGBT+ groups, Facebook, and a study website linked to all CBOs to reach potentially eligible participants.

#### Randomization

Participants will be randomized to the immediate intervention group or waitlist control group (12-week waitlist) at a 1:1 ratio, stratified by sex and gender [83] (cisgender men, cisgender women, transgender and gender nonbinary people), with a computer-generated sequence. Participants and researchers will not be blinded; in the informed consent process, potential participants will be told about the waitlist control.

#### Informed Consent

Immediately upon screening into the study, potential participants will be shown an informed consent form online and given time to read through it. Potential participants will be instructed to contact the study coordinator and provided with an email address if they wish to ask any questions or request clarifications before providing consent.

### Intervention

#### Overview

As there is no manualized intervention for COVID-19 prevention, we adapted efficacious eHealth interventions for HIV, the largest pandemic of the last century, by members of our research team [79,84]. The 3 primary outcomes—increasing COVID-19 knowledge, protective behaviors, and reducing psychological distress—are central to public health approaches to halt SARS-CoV-2 transmission [85,86].

#### Motivational Interviewing and Psychoeducation

The intervention builds on evidence-based eHealth interventions using motivational interviewing (MI) [87,88] and psychoeducation [89] approaches to increase health knowledge, behaviors, and reduce psychological distress [90-94]. Several MI-based studies have been conducted with LGBT+ people [95-97], including in India [79,84] and Thailand [98]. MI is a client-centered counseling approach that elicits and strengthens intrinsic motivation for change [87,99,100]. MI is based on Stages of Change theory, which enables tailoring to individual readiness for change with an emphasis on supporting client autonomy and volition [101,102]. Psychoeducation integrates education and counseling to promote mental health [89]. Consonant with MI, it is a strengths-based approach in which clients are considered partners in treatment [89]. Psychoeducational techniques are used to mitigate barriers to comprehending complex and emotionally laden information, with a focus on developing strategies to use the information proactively, such as in anticipating actions if one were to experience distress or loneliness [88].

#### Peer Counselor Training

Peer counselors will receive an initial 3-day online training along with a 2-day booster training immediately preceding the intervention. Training will be conducted by study coordinators and agency staff in each site, covering COVID-19, public health–recommended protective behaviors, pandemic stress (ie, anxiety, depression, social isolation), MI-based counseling, psychoeducation, and research ethics [78,103]. Training will include online small-group discussions, role-playing, and mock sessions, with peer counselor feedback also used to fine-tune the intervention.

#### Intervention Group

We use a 3-session peer-delivered MI-based brief counseling (45 minutes to 1 hour) format with weekly individual sessions, which has previously demonstrated effectiveness in interventions for alcohol, tobacco, and marijuana use, and HIV prevention [79,104-107]. Peer counselors will complete session-specific checklists (activities conducted, issues encountered, self-evaluated quality of engagement) following each session. To assess fidelity, supervisors will review a random selection of peer counselors’ initial sessions, which will be digitally recorded, and provide feedback using a structured checklist. Supervisors will conduct biweekly online group discussions to provide feedback, emotional support, and discuss and troubleshoot challenges to protocol implementation. In each online session, participants will complete a 4-item survey to evaluate content, satisfaction with the session and its duration, and any exposure to other interventions. Peer counselors write up brief counseling notes after each session, along with the brief self-evaluation. We use process evaluation to assess dose and implementation fidelity [107].

#### Waitlist Control Group

Governments and public health ministries in India and Thailand provide almost daily briefings about COVID-19 via multiple sources: TV, newspapers, Facebook, WhatsApp, LineChat, Instagram, and SMS text messages. Online messenger platforms (eg, WhatsApp, Line) provide LGBT-targeted information, with additional government mobile apps developed for general populations. The waitlist control group will receive brief reminders by mobile phone to support retention.

#### Assessments

Each participant will complete a baseline survey, a postintervention survey 2 weeks after their final eHealth session, and a follow-up survey 2 months after the postintervention survey. Waitlist controls will complete a second baseline survey immediately before beginning the eHealth intervention.

### Measures

#### Overview

Demographic data, including age, sex, gender identity, sexual orientation, city of residence, country of birth, education, and employment, will be obtained to determine the baseline equivalence of groups.

#### Primary Measures

##### Knowledge

COVID-19 knowledge [108,109] will be assessed using an index developed by the research team and based on published research [110,111].

##### Preventive Behaviors

Public health–recommended preventive behaviors [108,109] will be assessed using an index developed by the research team based on WHO and US Centers for Disease Control and Prevention guidelines.

##### Mental Health Measures

Depression and anxiety symptoms in the past 2 weeks will be measured using the PHQ-2 [112] and the Generalized Anxiety Disorder 2-item scale [113].

#### Secondary Measures

The indices assessed as secondary measures are listed in Textbox 1.

**Secondary measures.**

- UCLA Loneliness Scale [114]
- COVID Stress Scales (COVID danger, COVID traumatic stress) [115]
- Intended care-seeking [116]
- Unmet health care needs and perceived quality of care [117]
- COVID-19–related risk perception and testing (developed for this study)
- COVID-19 vaccines (developed for this study)
- Discrimination in Medical Settings Scale (DMS Scale) [118]
- Conspiracy beliefs [116,119-121]
- Attitudes toward government handling of COVID-19 [110]
- Support for government action regarding the pandemic [122]
- Alcohol Use Disorders Identification Test (AUDIT-C) [123]
- Sexual and reproductive health changes [124]
- Intimate partner violence changes [125]
- General and HIV-specific COVID-19 impacts [47,126]
- Household Water InSecurity Experiences (HWISE Scale) [127]
- Response to Stressful Experiences Scale (RSES-4) [128]
- Outness indicator [129]
- Technology ownership (developed for this study)

### Statistical Analysis Plan

Intervention efficacy will be assessed by comparing data from the second preintervention baseline survey from the waitlist control with postintervention data from the immediate intervention group. The *χ^2^* test for unadjusted analyses and logistic generalized estimating equations (GEEs) for adjusted analyses [130] will be used to assess dichotomous primary outcomes (COVID-19 knowledge, protective behaviors, psychological distress). Sensitivity analyses will use composite scores of primary outcomes with independent-samples *t* tests for unadjusted analyses and GEEs (Gaussian or Poisson) for adjusted analyses. To assess if intervention efficacy is sustained, waitlist control data from the second preintervention survey will be compared with intervention group follow-up data. We will use intention-to-treat analyses and also report per-protocol analyses [131]. In addition to analyses of efficacy for LGBT+ people as a whole, the sample size is powered for a priori subgroup analysis (cisgender gay/bisexual men, cisgender lesbian/bisexual women, transgender and gender nonbinary people). We will assess if intervention effects on protective behaviors are mediated by increases in knowledge, self-efficacy, perceived vulnerability, and decreases in conspiracy theories and psychological distress, and if the effects on psychological distress are mediated by resilience.

### Process Evaluation and Qualitative Analysis

Participant satisfaction and supervisor counseling session observation scores will be used to assess intervention satisfaction and fidelity, respectively. Dose will be determined by intervention session attendance. Post-RCT qualitative exit interviews will be conducted with purposive random samples [132,133] of select populations in each site, with selection criteria to be informed by survey data and focal populations of concern. These may include transgender and gender nonbinary persons, people living with HIV, and those with no intervention effect on reducing psychological distress (n=12 per group). In-depth interviews will be conducted online in accordance with methodological recommendations and ethical considerations amid a pandemic [134]. A semistructured interview guide will be used to explore experiences in the pandemic, focal challenges and strengths, identified supports, and thoughts about the intervention. Interviews will be audio-recorded, transcribed, translated into English, and examined using techniques from framework analysis [135] and thematic analysis [136] to explore pandemic-related challenges and resiliencies, population-specific and cross-group themes, perceived usefulness of the intervention, and intervention mechanisms.

## Results

This study was funded by the International Development Research Centre, Canada, from 2020 through 2021. Some of the development costs were funded by the Social Sciences and Humanities Research Council of Canada. The enrollment of participants began in August 2021. Baseline assessments, allocation, and intervention are currently underway. The first results are expected to be submitted for publication in 2022.

## Discussion

### Principal Findings

This protocol outlines the design of an RCT to evaluate the effectiveness of an eHealth peer intervention for increasing COVID-19 knowledge and preventive behaviors, and reducing psychological distress among sexual and gender minority people. To our knowledge, the proposed intervention is the first peer-delivered prevention program delivered via Internet of Things (IoT) devices (eg, PC, laptop, tablet, mobile phone) for LGBT+ people in LMICs amid the COVID-19 pandemic. If effective, it has the potential for widespread implementation at a relatively low cost, as it relies on peers and uses a delivery method that is both acceptable and accessible for LGBT+ people, including in an LMIC, during the pandemic.

### Strengths and Limitations

The key strengths of the proposed effectiveness study are the intervention’s focus on marshaling peer support among LGBT+ adults (aged 18 years or over) during the pandemic using digital technology. Mobile delivery using IoT devices means that the intervention is accessible during continuing stay-at-home orders, lockdowns, and waves of the pandemic, particularly in LMICs in which vaccines are not broadly available. The intervention is also adaptable for future pandemics and emergency situations. Engaging with others over online messenger platforms and apps is also comfortable and culturally appropriate for LGBT+ people, including those who may opt not to self-disclose their sexual orientation or gender identity in public forums to protect their privacy and safety in adverse familial and social environments. The intervention also addresses what may be population-specific concerns among LGBT+ people, who are often not included or considered in pandemic response planning or interventions designed for the general public. Further, the intervention can be accessed from home in relatively private spaces not requiring public attendance at LGBT-identified CBOs or services. The latter presents barriers due to stay-at-home orders, as well as more general obstacles for some LGBT+ people who are not “out” and for whom the risk of disclosure may present unacceptable “costs,” including loss of family support, job loss, harassment, and violence.

Another benefit of the intervention is its links to a broad spectrum of CBOs, both LGBT+-identified and non-LGBT+–identified. This means that CBOs could act as delivery partners to roll out the intervention if found to be effective. Relatedly, the intervention training and clinical supervision provided to peer counselors and counseling interns can act as a supportive mechanism during the pandemic for individuals from a vulnerable population, including CBO staff, as well as building capacity to address future emergency situations. Finally, the intervention was collaboratively designed by an international team with extensive experience in conducting research and providing health services to sexual and gender minorities in each country.

One limitation of the study is the reliance on participant self-report to collect data for the primary outcomes of this trial. Although this was chosen for feasibility and ethical considerations, and is a standard practice for psychosocial interventions, it is possible that measures of protective behaviors and mental health could be subject to underreporting or overreporting. To mitigate response bias and socially desirable responses, participants are reminded of the confidentiality of their responses at each survey occasion, are not asked for their names or home addresses, and are encouraged to be as honest as possible. Furthermore, the MI approach that guides the intervention is anchored in respect, lack of judgment, and acceptance of each participant’s current behaviors and perspectives; this milieu contributes to participants’ openness and honesty, and mitigation of socially desirable responses.

Due to time and budget constraints, the study was unable to provide tablets or smartphones to participants who did not own or have access to them. This may pose barriers to participation by individuals who do not have access to IoT devices or broadband internet. However, we used cross-platform programming with a responsive web design to ensure that the online content and eHealth sessions function and display correctly on a variety of devices, platforms, and screen sizes, including tablets and smartphones. Thailand [137] and India [138,139] have high rates of mobile phone penetration, both being among the top 20 countries in smartphone users in the world [140]. Further investigation will be needed to examine feasibility and efficacy among LGBT+ adults in rural areas, who may face more protracted challenges in a pandemic [141], as well as the potential impact of the gender gap in smartphone ownership, with women in LMICs being 20% less likely than men to own a smartphone or access the internet via a mobile device [142].

We mitigate threats to internal validity due to differential attrition by implementing a brief, 3-session, weekly intervention with 2-week immediate follow-up, which reduces the waitlist time for the control group. There is a reduced risk of contamination as an eHealth intervention for which participants will be individually recruited online and participate online. This threat is also mitigated due to stay-at-home orders and physical distancing guidelines that deter or prevent attendance at CBO sites, although guidelines may change during the course of the intervention.

Finally, the unpredictable course of the pandemic, and regional and local variation in severity and public health responses, has delayed onset of the study and created intermittent barriers and interruptions in implementation. Nevertheless, the development and testing of the intervention during a pandemic may increase its feasibility and external validity. Once developed, implemented, and tested, the intervention may be more readily usable for LGBT+ and other marginalized populations in future pandemics and other emergency situations.

### Conclusions

The development of a novel eHealth intervention designed for sexual and gender minority individuals to promote COVID-19 knowledge and protective behaviors, and reduce psychological distress represents an innovative approach to pandemic preparedness and response in real-world settings, including LMIC settings most severely impacted by the pandemic. The intervention protocol and materials will be linked and shared with existing CBOs and clinics serving sexual and gender minority populations, and if effective will be made publicly available, with the potential for broad implementation and a significant impact globally.

## Appendix

Multimedia Appendix 1 Four peer-review reports from the granting agency.

## Abbreviations

CBO: community-based organization
GEE: generalized estimating equations
IoT: Internet of Things
LGBT: lesbian, gay, bisexual, and transgender
LGBT+: LGBT and other persons outside of heteronormative and cisgender identities
LMIC: low- and middle-income country
MI: motivational interviewing
MSM: men who have sex with men
PHQ-2: Patient Health Questionnaire-2
RCT: randomized controlled trial
SDOH: social determinants of health
UNAIDS: Joint United Nations Programme on HIV/AIDS
WHO: World Health Organization
