# Supplementary material for: An eHealth Intervention for Promoting COVID-19 Knowledge and Protective Behaviors and Reducing Pandemic Distress Among Sexual and Gender Minorities: Protocol for a Randomized Controlled Trial (#SafeHandsSafeHearts)
Source: JMIR Res Protoc. 2021 Dec 10;10(12):e34381. doi: 10.2196/34381 (PMC8668022; doi:10.2196/34381)
Supplement: Multimedia Appendix 1 [file resprot_v10i12e34381_app1.pdf]

Review Type/Type d'évaluation: Committee Member 1/Membre de comité 1
Name of Applicant/Nom du chercheur: Newman, Peter Adam
Application No./Numéro de demande: 443836
Agency/Agence: CIHR/IRSC
Competition/Concours: 2020-05-12 Operating Grant: COVID-19 May 2020 Rapid Research Funding Opportunity/Subvention de fonctionnement : Possibilité de financement pour une intervention de recherche rapide contre la COVID-19 (mai 2020)
Committee/Comité: COVID-19 Rapid Research - Social Policy and Public Health Responses/Recherche rapide contre COVID-19- Rép. en matière de politique sociale & de santé publique
Title/Titre: An international multi-site, randomized controlled trial of a brief eHealth intervention to increase COVID-19 knowledge and protective behaviors, and reduce pandemic stress among diverse LGBT+ people

---

Assessment/Évaluation:

1. Quality of project:

Application responds to a number of the objectives in the call in that it is building on a well-established international network in Canada, India and Thailand and that it focuses the on experiences, needs, and priorities regarding COVID-19 and PHR protective measures among diverse LGBT+ persons. The need to focus on LGBT+ persons as a group who may be a higher risk of COVID-19 exposure and well as how they may experience amplified inequities or consequences due to governmental and societal responses is well justified. This research has a high likelihood of generating knowledge and evidence support COVID-19 public health interventions in Canada, other HIC and LMIC. The RCT is well described, especially for such a short, proposal and appears feasible. My only concern is the brief nature of the intervention and the short period of time before cross-over for the control group may make it challenging to detect a meaningful treatment effect. Sex- and gender-based analysis+ (SGBA+) is central to this proposal and is well integrated through-out.

2. Quality of Applicants:

The team for this application is impressive and diverse. It is clear that they have been working together effectively and have been able mobilize an existing network in creating this application. The degree of support from governmental organization and CBO is outstanding. I have no concern regarding the ability of the team to carry out the project.

3. Impact of the Research:

Applicants have demonstrated that they can execute a complex project in a short period of time, and have an existing KT network that will help disseminate findings. Crucially, the research will generate globally-relevant knowledge in improving COVID-19 response and PHR preventative measures for LGBT+ persons.

4. Budget:

Overall seems appropriate, but note that travel for the international meeting in Thailand may not be possible.

Review Type/Type d'évaluation: Committee Member 2/Membre de comité 2
Name of Applicant/Nom du chercheur: Newman, Peter Adam
Application No./Numéro de demande: 443836
Agency/Agence: CIHR/IRSC
Competition/Concours: 2020-05-12 Operating Grant: COVID-19 May 2020 Rapid Research Funding Opportunity/Subvention de fonctionnement : Possibilité de financement pour une intervention de recherche rapide contre la COVID-19 (mai 2020)
Committee/Comité: COVID-19 Rapid Research - Social Policy and Public Health Responses/Recherche rapide contre COVID-19- Rép. en matière de politique sociale & de santé publique
Title/Titre: An international multi-site, randomized controlled trial of a brief eHealth intervention to increase COVID-19 knowledge and protective behaviors, and reduce pandemic stress among diverse LGBT+ people

---

Assessment/Évaluation:

1. Quality of project:

a. Extent to which the application responds to the objectives;

The application is well aligned with the first objective of this funding opportunity.

b. Extent to which the application responds to one or more of the research areas;

The application is fit with the mental health issues related to with indirect consequence of COVID-19 pandemic.

c. Appropriateness of the proposed approach, study design, and populations;

This is an RCT with aim of understanding needs and challenges faced by diverse LGBT+ people in LMIC and HIC amid the COVID-19 pandemic and what is the level of COVID-19 knowledge, PHR protective behaviors, and pandemic related psychological distress. Also, to know if a brief, tailored, peer-delivered eHealth intervention will increase COVID-19 knowledge and protective behaviours, and reduce psychological distress among LGBT+ people.

The study is well designed with a strong team of researchers.

The sample size calculation is correct and the statistical analysis section is appropriate.

· Sex/gender considerations are well described.

**Review Type/Type d'évaluation:**	Committee Member 2/Membre de comité 2
**Name of Applicant/Nom du chercheur:**	Newman, Peter Adam
**Application No./Numéro de demande:**	443836
**Agency/Agence:**	CIHR/IRSC
**Competition/Concours:**	2020-05-12 Operating Grant: COVID-19 May 2020 Rapid Research Funding Opportunity/Subvention de fonctionnement : Possibilité de financement pour une intervention de recherche rapide contre la COVID-19 (mai 2020)
**Committee/Comité:**	COVID-19 Rapid Research - Social Policy and Public Health Responses/Recherche rapide contre COVID-19- Rép. en matière de politique sociale & de santé publique
**Title/Titre:**	An international multi-site, randomized controlled trial of a brief eHealth intervention to increase COVID-19 knowledge and protective behaviors, and reduce pandemic stress among diverse LGBT+ people

Assessment/Évaluation:

2. Quality of Applicants:

a. Track record of team members in fields related to the proposed research;

The applicants have excellent track records of research in the related fields.

If funded the team is quite capable of conducting the study.

3. Impact of the Research:

The research topic is well justified for the timely response to the global COVID-19 pandemic and can have a timely impact.

Clinical Trial:

This is multinational RCT and the team seems to be quite capable of networking capacity to conduct this multinational trail. The sample size is large enough to warrant generalizability of the results.

**Review Type/Type d'évaluation:**	Committee Member 3/Membre de comité 3
**Name of Applicant/Nom du chercheur:**	Newman, Peter Adam
**Application No./Numéro de demande:**	443836
**Agency/Agence:**	CIHR/IRSC
**Competition/Concours:**	2020-05-12 Operating Grant: COVID-19 May 2020 Rapid Research Funding Opportunity/Subvention de fonctionnement : Possibilité de financement pour une intervention de recherche rapide contre la COVID-19 (mai 2020)
**Committee/Comité:**	COVID-19 Rapid Research - Social Policy and Public Health Responses/Recherche rapide contre COVID-19- Rép. en matière de politique sociale & de santé publique
**Title/Titre:**	An international multi-site, randomized controlled trial of a brief eHealth intervention to increase COVID-19 knowledge and protective behaviors, and reduce pandemic stress among diverse LGBT+ people

Assessment/Évaluation:

Summary

The investigators proposed to conduct an international multi-site, randomized controlled trial of a brief eHealth intervention to increase COVID-19 knowledge and protective behaviors, and reduce pandemic stress among diverse LGBT+ people.

Strengths

1) Strong investigator team, multinational project, including India, Thailand and Canada.
2) The investigator team has track record in research in LGBT+ populations.
3) Design of the study reasonable.

Weaknesses

Aggressive timeline – we are already in June, and recruitment was supposed to start in May, but it is unclear if the team has adapted their e-Health interventions for HIV for COVID19. There are differences between HIV (primarily sexually/blood and body fluid transmitted) and COVID19 (primarily transmitted by droplets and respiratory route).

Overall

A strong team with a strong proposal that will help us understand whether the LGBT+ population is differentially affected by COVID19 and will help in the planning of further pandemic response in multiple countries.

Review Type/Type d'évaluation: Committee Member 4/Membre de comité 4
Name of Applicant/Nom du chercheur: Newman, Peter Adam
Application No./Numéro de demande: 443836
Agency/Agence: CIHR/IRSC
Competition/Concours: 2020-05-12 Operating Grant: COVID-19 May 2020 Rapid Research Funding Opportunity/Subvention de fonctionnement : Possibilité de financement pour une intervention de recherche rapide contre la COVID-19 (mai 2020)
Committee/Comité: COVID-19 Rapid Research - Social Policy and Public Health Responses/Recherche rapide contre COVID-19- Rép. en matière de politique sociale & de santé publique
Title/Titre: An international multi-site, randomized controlled trial of a brief eHealth intervention to increase COVID-19 knowledge and protective behaviors, and reduce pandemic stress among diverse LGBT+ people

---

Assessment/Évaluation:

Evaluation Criteria

1. Quality of project:

a. Extent to which the application responds to the objectives.

The proposed project responses to three objectives of this funding opportunity. First, the objective “to accelerate the availability of high-quality and real-time evidence to support Canada’s rapid response to the global pandemic” will be addressed by providing information about the efficacy of interventions (counselling and education) specifically tailored to LGBT+ people. Second, the objective “to enable Canadian engagement and coordination in national and international large-scale trials” will be addressed by leading an international RCT aiming to mitigate impacts of the pandemic on vulnerable groups (i.e., LGBT+ people). Third, the objective “to enhance local, national and international collaborative efforts, including in low- and middle-income countries, to mitigate the rapid spread of COVID-19” will be addressed in this project by providing information on variations in LGBT+ community responses to Covid-19 pandemic and the efficacy of specific preventive interventions.

b. Extent to which the application responds to one or more of the research areas.

The proposed project is relevant to “Social Policy and Public Health Responses” theme. It is currently not known how well the public health responses and policy measures in this pandemic address LGBT+ vulnerabilities, including differential risks by sex and gender. Interventions specifically tailored to LGBT+ will likely enhance health and wellbeing of this vulnerable population.

c. Addresses clear knowledge gap/need and high feasibility.

The proposed project addresses gaps in our knowledge of impact of the pandemic and associated public health measures on vulnerable groups, including LGBT+. It is not known if targeted interventions can help to mitigate some of the effects of Covid-19 pandemic in this population.
This is a large project; however, the team seems to be well connected and committed to support the project. This RCT will build on a previously funded project involving LGBT+ population.

d. Appropriateness of the proposed approach, study design, and populations.

The RCT is well designed. Phase 1 will include rapid qualitative research to adapt the eHealth interventions

**Review Type/Type d'évaluation:**	Committee Member 4/Membre de comité 4
**Name of Applicant/Nom du chercheur:**	Newman, Peter Adam
**Application No./Numéro de demande:**	443836
**Agency/Agence:**	CIHR/IRSC
**Competition/Concours:**	2020-05-12 Operating Grant: COVID-19 May 2020 Rapid Research Funding Opportunity/Subvention de fonctionnement : Possibilité de financement pour une intervention de recherche rapide contre la COVID-19 (mai 2020)
**Committee/Comité:**	COVID-19 Rapid Research - Social Policy and Public Health Responses/Recherche rapide contre COVID-19- Rép. en matière de politique sociale & de santé publique
**Title/Titre:**	An international multi-site, randomized controlled trial of a brief eHealth intervention to increase COVID-19 knowledge and protective behaviors, and reduce pandemic stress among diverse LGBT+ people

Assessment/Évaluation:

(originally for HIV) using a mixed-methods qual-QUANT design. Surveys from Ebola, N1H1, and SARS research will be also used. Phase 2 will include the RCT. Power calculations are provided.

Strengths:
1) Randomization to the immediate intervention group or the waitlist control group (with a crossover to receive the intervention later; July-Sept 2020 vs Oct-Nov 2020). This avoids ethical problems with respect to no-intervention control group.
2) Powered for subgroup analyses (cisgender gay/bisexual men, cisgender lesbian/bisexual women, transgender).
3) Assessment if intervention effects on public health recommended behaviours are mediated by increases in knowledge, self-efficacy, perceived vulnerability, decreases in conspiracy theories and psychological distress.
Weaknesses: this RCT is not blinded, however, this is due to the nature of the intervention that precludes blinding. Other limitations including differential attrition, contamination, and social desirability bias are addressed in the proposal.

e. Quality and appropriateness of the sex- and gender-based analysis+ (SGBA+). SGBA+ refers to the consideration and examination of sex, gender and other identity factors (e.g., age, race, ethnicity, culture, religion, geography, education, disability, income and sexual orientation) at all stages of the research process including planning and implementation of the research project and related activities – See How to integrate sex and gender into research.
a. See Appendix A: “Why Sex and Gender Need to be Considered in COVID19 Research” for information specific to this funding opportunity.

Sex, gender will be considered in the proposal as the RCT focusses on LGBT+ people.

f. For research involving Indigenous Peoples, appropriate consideration of TCPS2: Chapter 9 – Research Involving First Nations, Inuit and Métis Peoples of Canada, and demonstration of meaningful and culturally safe practices, plans and activities throughout the research project.

This research does not specifically target Indigenous Peoples. Indigenous Peoples are not excluded from the participation.

Review Type/Type d'évaluation: Committee Member 4/Membre de comité 4
Name of Applicant/Nom du chercheur: Newman, Peter Adam
Application No./Numéro de demande: 443836
Agency/Agence: CIHR/IRSC
Competition/Concours: 2020-05-12 Operating Grant: COVID-19 May 2020 Rapid Research Funding Opportunity/Subvention de fonctionnement : Possibilité de financement pour une intervention de recherche rapide contre la COVID-19 (mai 2020)
Committee/Comité: COVID-19 Rapid Research - Social Policy and Public Health Responses/Recherche rapide contre COVID-19- Rép. en matière de politique sociale & de santé publique
Title/Titre: An international multi-site, randomized controlled trial of a brief eHealth intervention to increase COVID-19 knowledge and protective behaviors, and reduce pandemic stress among diverse LGBT+ people

---

Assessment/Évaluation:

2. Quality of Applicants:

a. Track record of team members in fields related to the proposed research.

The team has an excellent track record of publications. The combined expertise includes prevention and intervention research with LGBT people, medicine, public health, epidemiology, social work, social science, and issues surrounding refugees and minorities.

b. Relevance and value-add of collaborations and partnerships to the research objectives.

International partnership will provide a unique international perspective on the effects of Covid-19 pandemic on LGBT+ community and possible differences in the efficacy of public health interventions. The current SSHHRC Partnership infrastructure includes Thai and Indian sites with HIV and STI trial activities.

c. Ability of the project team to carry out the proposed research, including project governance.

The team is able to carry out the project as intended. Expertise of the team, support from international sites, and the track record of publications is excellent, indicating that the project will be accomplished as planned.

d. Ability of team to quickly mobilize necessary resources, including by leveraging existing networks and /or research programs.

The team is well positioned to mobilize their current resources. International collaborations are established and team has expertise and experience to conduct this project.

e. For research involving Indigenous Peoples, extent to which the overall research team has the necessary knowledge, expertise and experience in Indigenous health research, and complementarity of expertise and synergistic potential to conduct Indigenous health research.

This research does not specifically target Indigenous Peoples.

3. Impact of the Research:

a. Clear justification for rapid response nature of project including the use of funding to achieve timely

**Review Type/Type d'évaluation:**	Committee Member 4/Membre de comité 4
**Name of Applicant/Nom du chercheur:**	Newman, Peter Adam
**Application No./Numéro de demande:**	443836
**Agency/Agence:**	CIHR/IRSC
**Competition/Concours:**	2020-05-12 Operating Grant: COVID-19 May 2020 Rapid Research Funding Opportunity/Subvention de fonctionnement : Possibilité de financement pour une intervention de recherche rapide contre la COVID-19 (mai 2020)
**Committee/Comité:**	COVID-19 Rapid Research - Social Policy and Public Health Responses/Recherche rapide contre COVID-19- Rép. en matière de politique sociale & de santé publique
**Title/Titre:**	An international multi-site, randomized controlled trial of a brief eHealth intervention to increase COVID-19 knowledge and protective behaviors, and reduce pandemic stress among diverse LGBT+ people

Assessment/Évaluation:

impacts and maximize health benefits;

Immediate benefits (months 1 – 8) will include training agency staff and promoting client protective behaviours to slow the spread of COVID-19 in Canada and in the participating sites. The results will provide important information to optimize public health responses to potential future waves of the spread of Covid-19.

b. Quality of the proposed knowledge translation plans to accelerate availability of high quality, real-time evidence for translation of research into policy, practice, and/or clinical guidelines to address the immediate response to the COVID-19 pandemic;

The KT plan is well developed. Continuous engagement of all sites with LGBT+ people will facilitate KT.

c. Potential to contribute to the global response to COVID-19.

International participation contributes to the global response, manly through the inclusion of community-based organizations focused on LGBT+.

4. Budget:

a. Appropriateness of the budget and justification for amount requested.
a. Reviewers may comment on the budget requested and make a formal recommendation, including clear and detailed rationale for any recommended budget cuts.
b. The applicant will receive the review as it is submitted by the reviewer.

The budget is appropriate.

Clinical Trials:

**Review Type/Type d'évaluation:**	Committee Member 4/Membre de comité 4
**Name of Applicant/Nom du chercheur:**	Newman, Peter Adam
**Application No./Numéro de demande:**	443836
**Agency/Agence:**	CIHR/IRSC
**Competition/Concours:**	2020-05-12 Operating Grant: COVID-19 May 2020 Rapid Research Funding Opportunity/Subvention de fonctionnement : Possibilité de financement pour une intervention de recherche rapide contre la COVID-19 (mai 2020)
**Committee/Comité:**	COVID-19 Rapid Research - Social Policy and Public Health Responses/Recherche rapide contre COVID-19- Rép. en matière de politique sociale & de santé publique
**Title/Titre:**	An international multi-site, randomized controlled trial of a brief eHealth intervention to increase COVID-19 knowledge and protective behaviors, and reduce pandemic stress among diverse LGBT+ people

Assessment/Évaluation:

All applications that include a clinical trial will also be evaluated on the following additional criteria (note that clinical trial research proposals have an extra page to account for the additional criteria):

a. Demonstrated, networked capacity to conduct multisite trials, including evidence that relevant trial groups/networks and/or sites are engaged.

Previous work of this team shows the capacity to conduct multisite trials.

b. Proposal provides evidence that the trial will be adequately powered and multi-site in order to have greatest potential for generalizability.

Statistical power calculations are presented.

c. Amplifies current clinical trial activities (nationally or internationally) or is ready to be implemented upon successful receipt of funds.

This project is well prepared to be implemented, the RCT will build upon existing SSHRC Partnership (MFARR-Asia, 2019-2026) network of investigators and community-based
organizations focused on LGBT+ inclusion.

d. Selection of appropriate outcomes with harmonized measures.

The international multisite trial will include harmonized data collection and transparent data sharing.
